# Internet Public Opinion Diffusion Mechanism in Public Health Emergencies: Based on Entropy Flow Analysis and Dissipative Structure Determination

**DOI:** 10.3389/fpubh.2021.731080

**Published:** 2021-10-15

**Authors:** Wanlian Li, Feng Zeng, Wei Zhou, Zhishao Chen

**Affiliations:** College of Public Administration and Law, Hunan Agricultural University, Changsha, China

**Keywords:** public health emergencies, internet public opinion, diffusion mechanism, entropy flow analysis, dissipative structure determination

## Abstract

As an empirical case, this study selected the illegal production process incidents of rabies and DPT (Diphtheria, Pertussis, Tetanus) vaccines by Changchun Longevity Biotechnology Co., Ltd., which occurred in July 2018. Based on the four factors involved in the spread of public opinion, the public health emergency, netizen, network media, and government, Brusselator model, and entropy method were applied to calculate the positive and negative entropy—to verify whether the Internet public opinion system is a dissipative structure. This study verified four evolution mechanisms in Internet public opinion diffusion, among which the trigger point of entropy-control occurred in the germination mechanism, the entropy-controlled disposal point occurred in the outbreak and fluctuating mechanism, and then became latency in the elimination mechanism. It provides a theoretical reference for the government to judge the stage of such diffusion and improve the governance ability of the opinion mentioned above.

## Introduction

In recent years, with the frequent occurrence of public health emergencies, research on Internet public opinion related to such events has also become an essential part of public health crisis management. The Internet public opinion describes the influence scope and disaster situation of public health events in the real space (1). Moreover, compared to traditional methods (systematic review and focus group study), analysis of online comments to news media reports is considered a faster and cheaper alternative while ensuring the breadth (2). On the other hand, if the relevant public opinion is not effectively supervised and guided in the transmission process, it is easy to cause serious social problems (3). Undoubtedly, social media has become the platform of the public to get information and express opinions and feelings (4).

Jo et al. (5) summarized and analyzed the public anxiety in the early stages of coronavirus disease 2019 (COVID-19) and evaluated the suitability of network information. They found a need to monitor public anxiety in the early stage of an outbreak. Furthermore, measuring public sentiment is essential for developing effective disease control policies (5). Using Twitter, Xiong et al. adopted sentiment analysis and a topic model to find that public opinion could help local governments with emergency responses (6). He et al. argue that the knowledge management framework of large-scale social media platform data should be constructed, and Internet public opinion should be monitored and analyzed using big data technology and knowledge management systems (7).

China has experienced an increasing number of public health emergencies in recent years, such as the illegal vaccine case of Shandong province, Ebola epidemic, African swine fever, hospitals in Hainan province selling fake HPV vaccines, 69 people infected with hepatitis C in a hospital in Jiangsu Province, and the COVID-19 outbreak. The Internet public opinion about such events is also spreading and developing rapidly in China, which can easily cause social panic. Meanwhile, it poses new challenges to effective handling of public health emergencies and maintaining an orderly and healthy Internet public opinion environment of the government.

With the largest Internet user population in the world, Internet public opinion of China is growing in influence. According to the 44th “Statistical Reports on Internet Development in China,” released by the China Internet Network Information Center (CNNIC) in August 2019, as of June 2019, netizens of China reached 854 million; network coverage reached 61.2% (8). The new media communication platform represented by Weibo, WeChat, Weishi, among others, has become the primary front for the spread of public opinion on public health emergencies. Because the new media platform is open, convenient, anonymous, and has a low threshold, it enables people to freely access information, express their opinions, and convey their views. This allows public opinion to be efficiently, truly, sharply and adequately reflected. At the same time, it is a real-time barometer and accurate direction indicator for government to screen the trend of public opinion on the Internet and understand it promptly.

The spread of Internet public opinion refers to an influential and biased opinion collection that consists of the emotions, attitudes, and opinions of the public on hot topics related to their own or social interests. This study is based on these four major factors involved in the spread of public opinion on public health emergencies: the public health emergencies, netizens, network media, and the government. By collecting data from public opinion-monitoring platforms, this study adopted entropy flow analysis to determine whether the Internet public opinion system forms a dissipative structure and then analyzed the diffusion mechanism of public health emergencies. This study has implications for providing theoretical references for a better understanding of public opinion evolution of public health events and improving public health crisis management. Existing literature mostly focuses on the causes, contents, and correct guidance of Internet public opinion. This study focuses on how to understand and judge the effective timing of public opinion governance and adopt the right means at the right time, which has certain contributions to the governance of Internet public opinions of similar events. However, this study selected a single typical case for analysis; in future research, comparative analysis of multiple cases can be carried out to enhance the rigor of research conclusions.

## Theoretical Framework and Literature Review

### Theoretical Analysis of Entropy Flow

Entropy is the amount of disorder in a system at any given time. Entropy theory defines disorder entropy as positive entropy *dS*_*A*_ and order entropy as negative entropy *dS*_*B*_ according to the different degrees of order and disorder of the system (9).

The total entropy of the system is composed of positive and negative entropy. The entropy generated within the system has irreversible characteristics and disorders for a single closed and isolated system. Thus, the positive entropy *dS*_*A*_ is formed spontaneously and is always greater than zero. The higher the entropy value of a single closed system, the worse the internal organizational ability of the system, and the greater and higher the degree of disorder. The smaller the entropy value, the stronger the internal organization ability, the smaller the degree of disorder, the higher the order degree. However, for a single-open system, the total entropy of the system is affected by the positive entropy *dS*_*A*_ and the negative entropy *dS*_*B*_. The positive entropy is the irreversible disordered entropy spontaneously formed in the system, which always has *dS*_*A*_ ≥ 0. The negative entropy is the ordered entropy flowing into the system from the outside, and its entropy value is negative, which can be canceled by the disordered entropy. With a decrease in the total entropy, the system evolves from disorder to order and stabilizes (10, 11).

In this study, the Internet public opinion diffusion system of public health emergencies is considered an open system composed of internal and external environments. The positive entropy is the disordered entropy formed spontaneously by public health emergencies and the internal environment of the Internet public opinion system composed of netizens, and its entropy value is positive. On the other hand, the negative entropy is the ordered entropy injected into the system by the external environment of the Internet public opinion system composed of government and network media. Furthermore, its entropy value is negative, which can counteract the disorder entropy. Thus, the Internet public opinion system of public health emergencies is an open system composed of the events themselves: netizens, network media, and the relevant government departments. Furthermore, its diffusion process is based on the condition of transforming from a disordered non-equilibrium state to an ordered equilibrium state and reaching the new ordered, stable system.

The expression of the total entropy change of the Internet public opinion system in public health emergencies is as follows:


(1) $$\begin{array}{l} dS=d_{A}S+d_{B}S \end{array}$$

where *d*_*A*_*S* is the irreversible entropy increase in the system, which belongs to the positive entropy. The disordered entropy spontaneously formed by the internal environment of the Internet public opinion system composed of public health emergencies and netizens, with constant *d*_*A*_*S* > 0. *d*_*B*_*S* is the entropy flow from outside the system, which belongs to the negative entropy. Finally, the ordered entropy injected into the system by the external environment of the Internet public opinion system is composed of network media and government, with constant *d*_*B*_*S* < 0.

The total entropy of the system can be positive and negative, and the positive and the negative are mainly affected by the information supply and demand gap between the information demand side (public health emergencies and netizens) and the information supply side (government and network media).

If the positive entropy input is greater than the negative entropy, it may lead to a positive total entropy value of the system. This indicates that the system is in a state of disorder and confusion; netizens still have demand for the truth regarding the event, and the process of Internet public opinion diffusion has not subsided. Contrastingly, if the positive entropy input is less than the negative entropy input, the total entropy value of the system may be negative. This indicates that the system is in an orderly and stable state, the netizens know the truth about the event, and the spread of Internet public opinion will gradually subside.

### Dissipative Structure Theory

Professor Prigogine, a Belgian scientist, first proposed the theory of dissipative structure in the 1960's. In this theory, the system changes from a disordered non-equilibrium state to an ordered equilibrium state by exchanging materials with outside. Finally, it forms a new ordered, stable structure known as a dissipative mechanism. The Brusselator model was proposed to conduct a quantitative analysis of the dissipative structure of systems (12). This method is widely used to verify whether a system has a dissipative structure (13). The Brusselator reaction sequence is as follows:


(2) $$\begin{array}{l} A\overset{k_{1}}{\to }X\\ B+X\overset{k_{2}}{\to }Y+D\\ Y+2X\overset{k_{3}}{\to }3X\\ X\overset{k_{4}}{\to }E \end{array}$$

where A and B are the initial reactants of the Internet public opinion system, D and E are the reaction products of the system, and they remain the same. X and Y are intermediate products of the system, and their amount varies with the length of the opinion diffusion time. The Brusselator diffusion dynamics model is expressed as follows:


(3) $$\begin{array}{l} \frac{d_{x}}{d_{t}}=k_{1}A-\left(k_{2}X+k_{4}\right)X+k_{3}X^{2}y\\ \frac{d_{y}}{d_{t}}=K_{2}BX-k_{3}x^{2}y \end{array}$$

If *A* > 0 and when *B* > 0, it represents the exchange of information between the external environment and the system; the expression has a unique equilibrium point *X* = *A, Y* = *B*/*A*, with the critical condition *B* > 1 + *A*^2^. Thus, the model can analyze the positive and negative entropy of Internet public opinion system.

Applying the Brusselator model, A and B are defined as the positive and the negative entropy flows, respectively, making the Internet public opinion diffuse. D and E represent the two states when the four factors in the system diffuse. D is a non-dissipative structure, E is a dissipative structure, and X and Y are intermediate state I and intermediate state II of the diffusion process, respectively (14).

According to the equation and inference of the Brusselator model, only when *B* > 1 + *A*^2^ does the system produces self-organization behavior. When the input of negative entropy B in the external environment is small, and the critical condition is not reached, the positive entropy plays a leading role in the system. Additionally, the diffusion of Internet public opinion does not become a dissipative structure, which indicates that the diffusion still tends to be chaotic and disordered. When negative entropy B is input, the external environment reaches the critical value; the system is in a crucial state, which indicates that the system and the external environment no longer exchange information and tend to be stable. Suppose the input of negative entropy B in the external environment exceeds the critical value. In that case, the system will become a dissipative structure, which indicates that the diffusion of public opinion in the network will gradually and steadily develop and progressively dissolve. Based on the formula |*B*| > 1 + *A*^2^, the criterion of the dissipative structure of the diffusion process of the system is deduced. The expression is as follows:


(4) $$|B|>(1+A^{2})\{\begin{array}{l} >0,\text{ reaches dissipative structure}\\ =0,\text{ reach criticalstate}\\ <0,\text{ nondissipative structure} \end{array}$$

According to the above expression, in the process of public opinion diffusion in public health emergencies, as long as the concentration of positive and negative entropy A and B is controlled and the conditional relationship |*B*| > 1 + *A*^2^ is reached, the system will evolve from imbalance to order.

### Crisis Life Cycle Theory

The public opinion diffusion process has its life cycle. The process of dividing the life cycle of the evolution of public opinion is based on crisis life cycle theory. Fink first proposed a four-stage theoretical model of public crisis communication, which divided the crisis life cycle into prodromal (the warning stage), acute (when the crisis occurs), chronic crisis stage (the cleanup stage), and crisis resolution stage (15). In 1992, Pauchant and Mitroff proposed a five-stage theoretical model, which indicates the following: detection of the crisis (warning signs), prevention/preparation of the crisis (prepare for its occurrence), containment (efforts to limit the duration of the crisis and localize it), recovery (restoring order), and learning (evaluation and examination) (16).

Coombs proposed a three-stage model comprising the precrisis (actions that organizations perform before a crisis), crisis (recognition, containment, and business resumption), and postcrisis stages (prepared for the next crisis and make a positive impression of the management abilities of the government) (17). He et al. investigated the online opinions of young college students and divided the online public opinion diffusion process into four stages: prodromal, outbreak, fluctuating, and fading stages (18). Zhao et al. found that the attention of the public during the COVID-19 epidemic can be divided into three stages based on their study of Sina Microblog hot search list: incubation period (low and unstable public attention), epidemic period (concentrated increase), and widespread period (continued public attention) (19).

Public health emergencies are public crisis events, and public opinion diffusion in public health networks is similar to that of crisis information dissemination. Therefore, this theory has essential reference and guiding value for studying the period of Internet public opinion diffusion.

In summary, this study makes the following deductions. First, at the beginning of the event, due to the sensitivity of the event and the urgent needs of netizens, the original balance of the Internet public opinion system was broken, which prompted the germination stage, and public opinion began to spread. Then, as the external negative entropy of the system gradually increases, and when the absolute value of the negative entropy is greater than the positive entropy value, public opinion enters the stage of elimination. Finally, the system reaches a dissipative structure.

**Figure 1:**
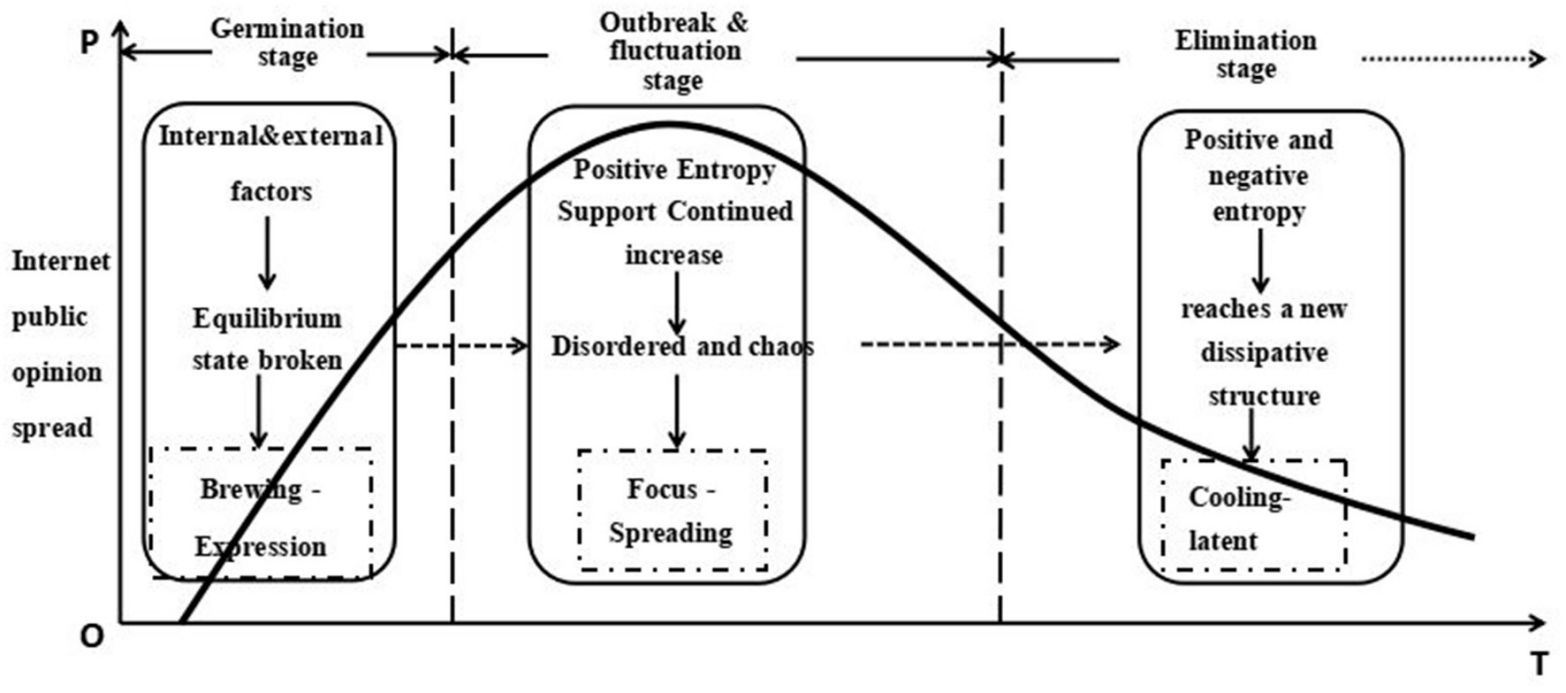
The process of dissipative structure formation of Internet public opinion diffusion.

The total entropy change of the system shows that the spread of Internet public opinion of this event has undergone a process of order before germination, disorder during germination, chaos during an outbreak and fluctuation, and gradually formed an orderly and stable development in the final elimination stage. The process of dissipative structure formation of Internet public opinion diffusion in public health emergencies is illustrated in Figure 1.

## Methodology and Measurement

### Positive and Negative Entropy Index

The main body of the internal environment of public opinion systems for public health emergencies is public health events and netizens, which are the primary sources of the positive entropy.

“*Event entropy”* is reflected by the heat of Internet public opinion on public health emergencies, expressed as the degree of gathering such opinion and the change in public opinion pressure. Due to the initial outbreak of public health emergencies, the public urgently needs the truth regarding the event and the handling of the event, so the “*netizen entropy”* is generated. Therefore, the amount of reading, commenting, forwarding, and likes can be expressed as netizen entropy.

The media and government are the main bodies of the external environment of the system. The negative entropy of the system is mainly composed of the “*media entropy”* and the “*government entropy.”* Therefore, the system should be promoted from disorder to orderly development.

“*Media entropy”* refers to the amount of media tracking, reporting, and spreading information on public health emergencies, which can be reflected by the media index and the amount of media reporting at the central level. “*Government entropy”* is the information publicity and handling behavior of relevant government departments such as the State Council, the provincial and municipal healthcare commission. It mainly releases information through web portals, so the release of the government plays an essential role in guiding the spread of Internet public opinion of the event, which can fully reflect the entropy of the government. The notification and disposal of the event of the government were counted from the horizontal diffusion (regions) and vertical diffusion (government level) to serve as the assignment of government release.

The positive and negative entropy index system of the Internet public opinion system of public health emergencies is presented in Table 1.

**Table 1:** Positive and negative entropy index system of internet public opinion system.

**Target layer**	**Elements layer**	**Variable layer**	**Interpretation**
Positive entropy A	Event entropy	A1 Event heat value	The increase in the impact index of the event.
	Netizen entropy	A2 Search Volume	The weighted sum of the search frequency of the core keywords of the Baidu search engine, indicating the degree of the netizen's concern about the public health emergency and its changes.
		A3 Net behavior	The weighted sum of the number of participating behaviors such as search, comment, likes, forwarding and reading of event keywords.
Negative entropy B	Media entropy	B1 Media index	Except for the central media, all the news titles containing the keywords of the event in the reports of the major new media network platforms were collected by Baidu News.
		B2 Central media coverage	Number of articles reported by 18 central news organizations (People's Daily, Xinhua News Agency, Central Radio and Television Station, Qiushi magazine, PLA newspaper, Guangming Daily, Economic Daily, China Daily, Science and Technology Daily, CPPCC newspaper, China Discipline Inspection and Supervision newspaper, Learning Times, Workers Daily, China Youth Daily, China Women's Daily, Farmers Daily, Legal Daily, China News Agency) during the period of the event.
	Government entropy	B3 Government release	According to “ZhiWeiData,”^a^ the top nine regions in terms of netizens' attention were obtained, and the number of official reports and disposal information of crisis events on the portals of governments at all levels in these regions were collected. Assignments to central, provincial, municipal, county, and township governments based on 5, 4, 3, 2, and 1, respectively^b^. (Figure 2 shows the top nine regions with netizens' attention)

a *ZhiweiData is a famous social media data service company in China. With massive Internet information platform data, ZhiweiData provides multilevel services of artificial intelligence and big data technology, intelligence perception, intelligence analysis, and intelligence think tank*.

b *As shown in Figure 2, the top nine regions with the attention of netizens are Jiangsu, Guangdong, Zhejiang, Shandong, Beijing, Shanghai, Sichuan, Henan, and Shaanxi, with 20.9, 19.6, 18.5, 15.9, 14.8, 7.7, 0.3, 0.2, and 0.2%, respectively. Using “Changchun Longevity Biotechnology Co., Ltd., Vaccine” as the key search term, at the official website of the Health and Health Commission Portal in the above nine provinces and municipalities, the amount of information published within the period of the event was counted. The number published was multiplied by the number of government rankings to obtain the specific value*.

**Figure 2:**
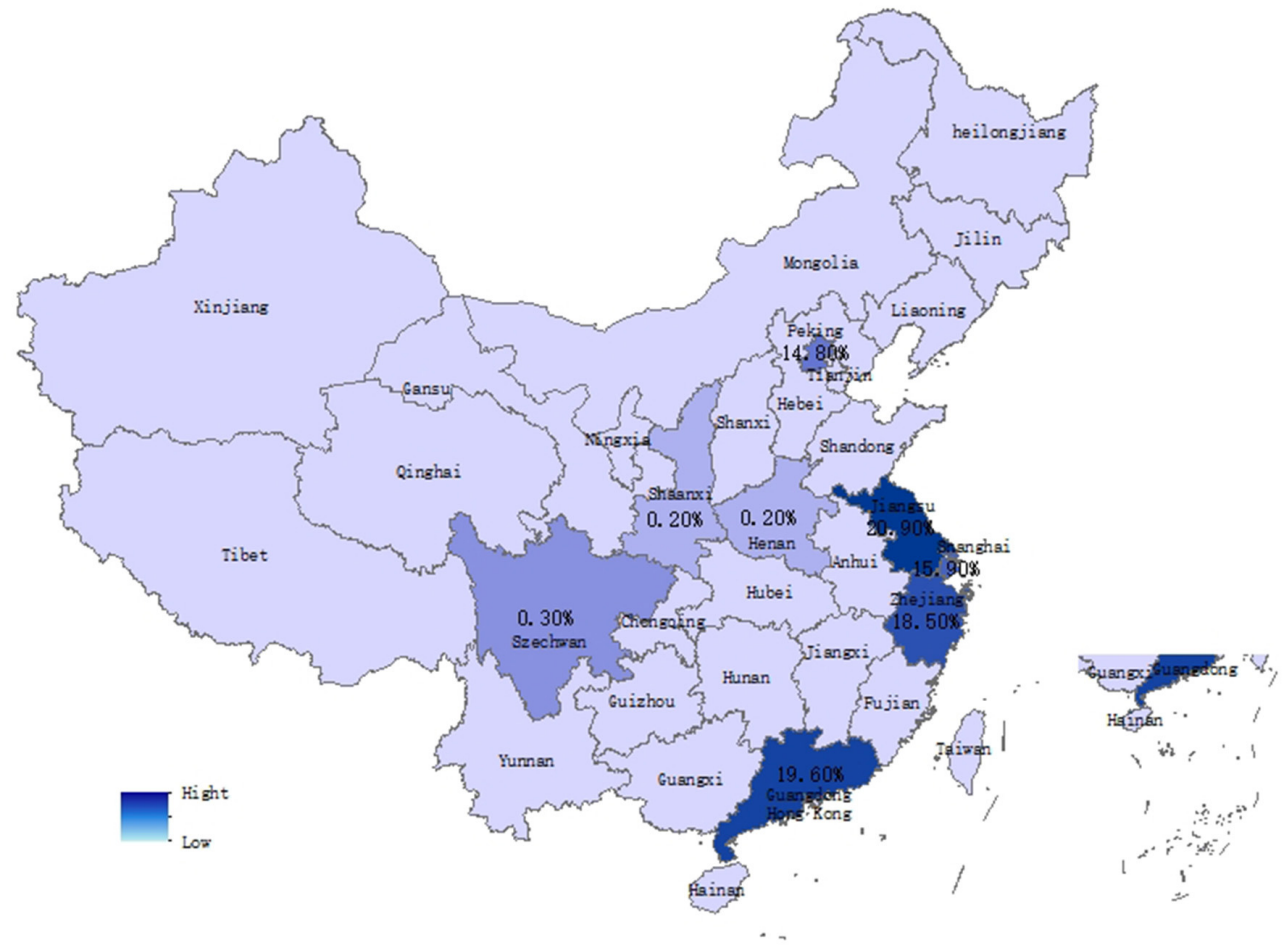
Regional distribution of netizens involved in Changchun Longevity Biotechnology Co., Ltd., vaccine fraud incident (www.zhiweidata.com). The nine provinces marked with numerical values are the most active netizens in this public opinion event, and the numerical values represent their proportion in the top nine.

### Data Processing

Due to the difference in dimensionality, quantity grade, and utility of the positive and negative entropy index, the extremum standardization method is used to make the data dimensionless. It is necessary to shift the data to avoid an invalid logarithm of negative entropy. *X*_*ij*_ is the value of the index *j* in the stage *i*(*i* = 1, 2, …*m*; *j* = 1, 2, …*n*). The normalized value *Z*_*ij*_ after normalization is expressed as follows:

When *X*_*ij*_, it is a positive entropy indicator:


(5) $$\begin{array}{l} Z_{ij}=1+\frac{X_{ij}-\min\left(X_{1j},X_{2j},\ldots X_{mj}\right)}{\max\left(X_{1j},X_{2j},\ldots X_{mj}\right)-\min\left(X_{1j},X_{2j},\ldots X_{mj}\right)} \end{array}$$

When *X*_*ij*_, it is a negative entropy indicator:


(6) $$\begin{array}{l} Z_{ij}=1+\frac{\max\left(X_{1j},X_{2j},\ldots X_{mj}\right)-X_{ij}}{\max\left(X_{1j},X_{2j},\ldots X_{mj}\right)-\min\left(X_{1j},X_{2j},\ldots X_{mj}\right)} \end{array}$$

### Model Operations

#### Calculation of Positive Entropy in Internet Public Opinion System

The Changchun Longevity Biotechnology Co., Ltd., vaccine fraud period was from July 15, 2018, to August 10, 2018. Based on the critical nodes of events, the diffusion process of event Internet public opinion is divided into four stages: germination stage X1 (July 15 to July 22), outbreak stage X2 (July 23 to July 26), fluctuation stage X3 (July 27 to August 8), and elimination stage X4 (August 9 to August 10). Thus, the proportion of each indicator of the index system of the positive entropy flow presented in Table 1 (*m*_*A*_ = 4, *n*_*A*_ = 3) can be calculated as follows:


(7) $$\begin{array}{l} PA_{ij}=\frac{xA_{ij}}{\overset{4}{\underset{j=1}{\sum }}xA_{ij}},\left(j=1,\ldots ,3\right) \end{array}$$

Make $K_{A}=\frac{1}{\ln\;4}$, then the entropy value of each index in the positive entropy flow index system is


(8) $$\begin{array}{l} e_{Ai}=-\frac{1}{\ln\;4}\overset{4}{\underset{j=1}{\sum }}p_{A_{ij}}\ln\;p_{A_{ij}},\left(j=1,\ldots ,3\right) \end{array}$$

The above equation satisfies 0 ≤ *e*_*Aj*_ ≤ 1, and the information entropy redundancy (difference coefficient) of each index is


(9) $$\begin{array}{l} {\text{g}}_{Aj}=1-e_{Aj} \end{array}$$

The weight of the index *i* can be obtained from the different coefficients of each index:


(10) $$\begin{array}{l} W_{Aj}=\frac{g_{Aj}}{\overset{4}{\underset{j=1}{\sum }}g_{Aj}} \end{array}$$

The positive entropy of the Internet public opinion system is


(11) $$\begin{array}{l} E_{A}=\overset{4}{\underset{j=1}{\sum }}\lambda_{Aj}e_{Aj} \end{array}$$

#### Calculation of Negative Entropy Value in Internet Public Opinion System

For the negative entropy flow index system presented in Table 1 (*m*_*B*_ = 4, *n*_*B*_ = 3), the proportion of each index is


(12) $$\begin{array}{l} P_{B\text{ij}}=\frac{x_{Bij}}{\overset{4}{\underset{j=1}{\sum }}x_{Bij}},\left(i=1,\ldots ,3\right) \end{array}$$

Make $K_{B}=\frac{2}{\text{ln}\;4}$, then the entropy of each index in the negative entropy system is:


(13) $$\begin{array}{l} e_{Bi}=\frac{2}{\ln\;4}\overset{4}{\underset{j=1}{\sum }}p_{Bij}\ln\;p_{Bij},\left(i=1,\ldots ,3\right) \end{array}$$

The above equation satisfies −2 ≤ *e*_*Bi*_ ≤ 0, and the different coefficient of each index is


(14) $$\begin{array}{l} {\text{g}}_{Bi}=2+e_{Bj} \end{array}$$

According to the different coefficients of each index, the weight of the index *i* can be obtained as follows:


(15) $$\begin{array}{l} W_{Bj}=\frac{g_{Bj}}{\overset{4}{\underset{j=1}{\sum }}g_{Bj}} \end{array}$$

The negative entropy of the Internet public opinion system is


(16) $$\begin{array}{l} EB=\overset{4}{\underset{j=1}{\sum }}\lambda_{Bj}e_{Bj} \end{array}$$

According to the weight of each index, the comprehensive score of the spread of Internet public opinion in each stage can be obtained as follows:


(17) $$\begin{array}{l} S_{i}=\overset{m}{\underset{j=1}{\sum }}W_{j}*P_{ij} \end{array}$$

The test formula for the calculation results is as follows:


(18) $$\begin{array}{l} W_{j}=W_{Aj}+W_{Bj}=1 \end{array}$$

If *W*_*j*_ is not 1, it indicates that the calculation result is incorrect.

#### Determination of the Dissipative Structure of Internet Public Opinion System

The essence of the evolution process that emphasizes the transformation of a disordered state to an ordered state in a dissipative structure system is to inject ordered negative entropy flow in time and reduce the generation of positive entropy flow. For example, the Internet public opinion system of public health emergencies is an open system formed by the interaction of four factors (public health emergency, netizens, social media, and government departments). It transforms from a disordered non-equilibrium state to an ordered equilibrium state. Furthermore, it achieves a new entropy change process of an ordered and stable system, which meets the requirements of the judgment model of the dissipative structure.

Therefore, according to the classical Brusselator diffusion dynamics model (12), the positive and negative entropy flow system of the Internet public opinion system for public health emergencies is calculated. Moreover, the positive and negative entropy values of its diffusion process are obtained. According to the criterion, the entropy value calculation results are substituted into equation (4) to determine whether the diffusion reaches the dissipative structure.

## Empirical Study

### Case Description

**Figure 3:**
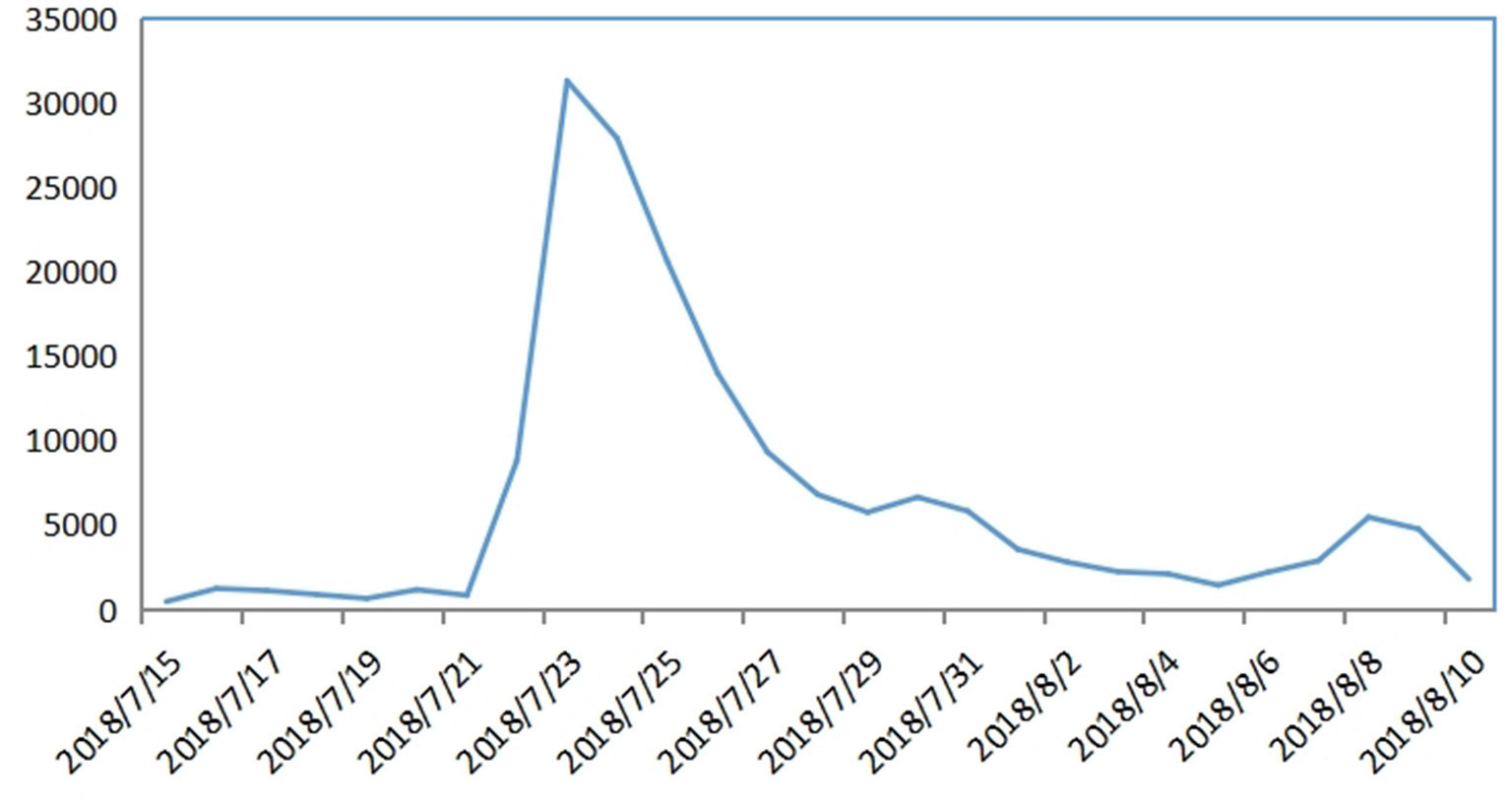
Changchun Longevity Biotechnology Co., Ltd. vaccine fraud incident: Internet public opinion development trend (source: ZhiWeiData). The vertical axis represents the number of pieces of information; the horizontal axis represents the date.

As an empirical case, this study selected the illegal production process incidents of rabies and diphtheria, pertussis, tetanus (DPT) vaccines by Changchun Longevity Biotechnology Co., Ltd., which broke out in July 2018. According to the critical nodes of public opinion development (Figure 3), the diffusion process of event Internet public opinion is divided into four stages: germination stage X1 (July 15 to July 22), outbreak stage X2 (July 23 to July 26), fluctuation stage X3 (July 27 to August 8), and elimination stage X4 (August 9 to August 10).

The Internet public opinion spread situation regarding the Changchun Longevity Biotechnology Co., Ltd., vaccine fraud can be roughly divided into anger and pain (germination), accountability (outbreak and fluctuation), disposal, and reflection (elimination).

#### Internet Public Opinion Germination: Anger and Pain

The period during which Internet public opinion was triggered was July 15 to 22, 2018; the public expressed anger and sad emotional tendencies, with an upward trend. The State Drug Administration circulated a notice about the company regarding falsified records and the illegal production of vaccines on July 15, 2018. Although the move did not attract wide public attention, people began to express anger and sadness on major social media platforms.

On July 16, “Guangming Daily,” “Beijing News,” “People's Daily,” “CCTV News,” and other relevant authoritative central media began to report the incident and reprint the official response of the government. Thus, the Internet public opinion field was constantly developing.

On July 17, ZAKER News (a well-known Chinese information publishing platform) first released a report that employees of Changchun Longevity Biotechnology Co., Ltd., were under the “seal fee,” which has long been an indicator of suspected criminal activity. Subsequently, “China News Network” and other media sources reprinted the article.

On July 19, the Food and Drug Administration of Jilin province fined Changchun Longevity Biotechnology Co., Ltd., 3.4429 million RMB for the illegal production of vaccines. As a result, the focus shifted to how government departments deal with such matters.

On July 20, after a review article published by the Procuratorial Daily (a media platform hosted by the Supreme People's Procuratorate of the People's Republic of China), the relevant Internet public opinion volume increased again. Major network platforms such as Weibo and WeChat continued to publish updates, and many netizens were involved in forwarding and following.

On the evening of July 21, an article released from We-Media titled “King of Vaccines” triggered shock and panic, exposed the scandals of jerry-building, fraud, evasion of supervision, low antigen content of vaccine, and many more. Furthermore, it revealed that a batch of about 250,000 doses of DPT vaccine produced by Changchun Longevity Biotechnology Co., Ltd., that had failed tests had been sold to Shandong province.

On July 22, Prime Minister Keqiang Li gave instructions on the vaccine fraud incident. Internet public opinion began to soar, causing netizens to continue paying attention to and discussing the public health emergency. Additionally, the volume of network public opinion on various social platforms also peaked on the same day.

#### Outbreak and Fluctuation of Internet Public Opinion: Accountability

On July 23 and 26, 2018, Internet public opinion continued to erupt and increase in volume. The government's handling of the incident and the health of vaccinated children has become a spotlight of public attention. Therefore, the overall emotional tendency of netizens was mainly related to accountability.

Following the instructions of the Prime Minister on July 22, “people.cn,” “China National Radio,” and other central media reprinted the instructions, and incident-related Internet public opinion continued to “heat up” in tone.

On July 23, General Secretary Jinping Xi issued a vital directive, stressing that all government departments should attach great importance to the incident, immediately file a case for investigation, determine the truth regarding the incident, be seriously accountable, and handle it per laws and regulations. Consequently, the volume of related public opinion on WeChat, Weibo, and other major social platforms peaked.

On July 24, the Commission for Discipline Inspection of Jilin province investigated the corruption in the Changchun Longevity Biotechnology Co., Ltd., vaccine fraud case. It detained the chairman per the law.

On July 25, the flow and the control of fraudulent vaccines were reported by the State Drug Administration.

On July 26, the former party secretary and director of Jilin Provincial Food and Drug Administration was prosecuted for bribery.

#### Elimination of Internet Public Opinion: Disposal and Reflection

Those involved were investigated and punished, and the topic “vaccine fraud incident” gradually became less of a hot topic. Subsequently, netizens began to reflect more on whether such fraud would be repeated. However, from July 27 to August 8, 2018, the follow-up of “where the vaccine in question went” and the quality of the vaccine were still critical points of public concern.

On July 27, the State Council ascertained the facts and issued a circular.

On August 6, the State Council investigation team announced its progress regarding its investigation of the illegal production of rabies vaccines at Changchun Longevity Biotechnology Co., Ltd.

On August 8, the government introduced a rabies vaccine reinsertion program.

### Entropy Value Calculation and Determination of the Dissipative Structure

#### Positive and Negative Entropy Flow Calculation

The original values of the positive and negative entropy indexes in four stages (germination, outbreak, fluctuation, and elimination) are reported in Table 2. As the dimensionality of each index is different, this study uses the extreme value standardization method to conduct the dimensionless processing of each index and continues the standardization calculation to the initial value according to formulas (1) and (2). The calculation results are presented in Table 3.

**Table 2:** Original values of positive and negative entropy index.

	**Positive entropy index A**			**Negative entropy index B**		
	**A1**	**A2**	**A3**	**B1**	**B2**	**B3**
X_1_	15,009	62,129	25,126,080	−6,149	−22	−23
X_2_	93,666	138,895	153,704,453	−15,066	−64	−85
X_3_	56,707	51,502	56,472,512	−6,261	−63	−70
X_4_	6,508	3,365	1,423,073	−1,790	−11	−25

**Table 3:** Normative values of positive and negative entropy index.

	**Positive entropy index A**			**Negative entropy index B**		
	**A1**	**A2**	**A3**	**B1**	**B2**	**B3**
X_1_	0.187781959	0.490227994	0.240087424	0.395938894	0.286792453	0.1
X_2_	1	1	1	1	1	1
X_3_	0.618358613	0.419658378	0.425348346	0.400771383	0.983018868	0.782258065
X_4_	0.1	0.1	0.1	0.1	0.1	0.129032258
	1.906140572	2.009886372	1.76543577	1.898598976	2.369811321	2.25625

This study obtained the *P* matrix of positive and negative entropy of the Internet public opinion system of the Changchun Longevity Biotechnology Co., Ltd., vaccine event (Table 4) using the formula (3). Based on the *P*-value, the contribution degree of the positive and negative entropy indexes in the germination, outbreak, fluctuation, and elimination stages were calculated (Table 5).

**Table 4:** *P* matrix.

	**Positive entropy index A**			**Negative entropy index B**		
	**A1**	**A2**	**A3**	**B1**	**B2**	**B3**
X_1_	0.098514224	0.243908313	0.135993293	0.208542667	0.121019108	0.04432133
X_2_	0.52462028	0.497540564	0.566432388	0.526704171	0.421974522	0.443213296
X_3_	0.324403468	0.208797066	0.24093108	0.211087959	0.414808917	0.346707175
X_4_	0.052462028	0.049754056	0.056643239	0.052670417	0.042197452	0.057188812

**Table 5:** Total contributions.

	**Positive entropy index A**			**Negative entropy index B**		
	**A1**	**A2**	**A3**	**B1**	**B2**	**B3**
X_1_	0.228312067	0.344145578	0.271326979	−0.32691391	−0.255568979	−0.138118082
X_2_	0.338422341	0.347322216	0.321958785	−0.33767859	−0.364083981	−0.360644496
X_3_	0.365202805	0.327058153	0.342903801	−0.32834318	−0.365005841	−0.36725815
X_4_	0.154640518	0.14929517	0.162621756	−0.15504598	−0.133571623	−0.163639896
	1.086577731	1.167821118	1.098811321	−1.14798165	−1.118230424	−1.029660623

Based on formula (4), Table 5 indicates that the most significant contribution is from the search volume A2 and the media index B1 in the germinating stage. The contribution of the government release B3 is lower. In the outbreak stage, the event heat value A1, the search volume A2, the net behavior A3 in the positive entropy flow, and the central media coverage B2. Furthermore, the government release B3 in the negative entropy flow contributed largely, indicating that netizens were eager for the truth about the event and expected the government to explain to the public.

In the fluctuation stage, the contribution of the event heat value A1, the net behavior A3, the central media coverage B2, and the government release B3 is large. This indicates that, after the outbreak stage, the internal demand in the Internet public opinion system is still larger than the external supply. The government still has not dealt with the event effectively, which leads to the continued spread of the related Internet public opinion, and the event continues to develop.

During elimination, the event heat value A1, search volume A2, and central media coverage B2 decreased. This indicates that, after the fluctuation stage, the relevant government departments dealt with the events reasonably and scientifically to meet the interests of the public. As the heat of the event subsided, netizens were gradually in a “wait-and-see” state. At this time, the system had reached an orderly equilibrium state where supply was greater than demand.

Using the positive entropy flow formulas (4–6), and (7) and the negative entropy flow formulas (9–11), and (12), the entropy *E*_j_, difference coefficient *D*_j_, weight *W*_j_, and total entropy *E* of the system can be obtained, respectively (Table 6). According to the entropy value *E* of each index, in the course of the Internet public opinion diffusion of the vaccine fraud, the event heat value A1 and the net behavior A3 input the most positive entropy flow to the system, with 0.093 and 0.090, respectively. This indicates that the event heat and the likes, comments, and forwarding behaviors of the netizens are the core forces to promote the accumulation of public opinion entropy in the network. On the other hand, the government release and central media report input the most negative entropy flow into the system, with −0.419 and −0.342, respectively. This indicates that the active involvement of government departments and authoritative central media reports is a significant force to control the disorderly development of the positive entropy flow of Internet public opinion and to realize the system toward an orderly and balanced state. According to the results *W*_j_, in the positive entropy flow of the system, the weight of event heat value A1 and the net behavior A3 is about 0.118 and 0.113, respectively. This indicates that the heat of public health emergencies and the information generated by likes, comments, and retweet behaviors of the netizens significantly influence the positive entropy index of the system. In the negative entropy flow of the system, the weights of central media coverage B2 and government release B3 are about 0.212 and 0.282, respectively. This indicates that the active reporting of the central media and the timely, scientific, and effective intervention of the government can effectively control the spread of Internet public opinion, promote the system to reach a new equilibrium state, and have a significant influence on the negative entropy index of the system.

**Table 6:** Entropy values and weight of the index.

	**Positive entropy index A**			**Negative entropy index B**		
	**A1**	**A2**	**A3**	**B1**	**B2**	**B3**
e_1_	0.164692343	0.24824856	0.195721044	−0.47163707	−0.368708099	−0.199262273
e_2_	0.244120117	0.250540019	0.232244171	−0.48716723	−0.525262154	−0.520300025
e_3_	0.263438138	0.235922588	0.247352807	−0.47369907	−0.526592116	−0.529841511
e_4_	0.111549554	0.107693701	0.1173068	−0.22368406	−0.192703118	−0.236082466
*E*_j_	0.783800152	0.842404868	0.792624822	−1.65618744	−1.613265487	−1.485486275
*D*_j_	0.216199848	0.157595132	0.207375178	0.343812559	0.386734513	0.514513725
*W*_j_	0.118385819	0.086295291	0.113553643	0.188263461	0.211766487	0.281735299
*E*	0.092790823	0.072695573	0.090005436	−0.31179958	−0.341635564	−0.41851392
Total entropy	0.255491833			−1.071949063		
|*B*| − (1 + *A*^2^)	0.006672987					

Based on the formula (13), the comprehensive scores of the four stages (germination, outbreak, fluctuation, and elimination) of Internet public opinion diffusion regarding the vaccine fraud event are presented in Table 7. The comprehensive scores for the four stages are 0.13, 0.48, 0.67, and 0.05, respectively, indicating that the negative entropy is the largest in the fluctuation stage, followed by the outbreak, germination, and elimination stages.

**Table 7:** A comprehensive score of internet public opinion diffusion.

	**X_**1**_**	**X_**2**_**	**X_**3**_**	**X_**4**_**
Comprehensive	0.125528999	0.482751513	0.667113242	0.051900375
score				
Rank	3	2	1	4

#### Dissipative Structure Determination

Table 6 indicates that the positive entropy A of Internet public opinion diffusion in the vaccine fraud event is 0.255, while the negative entropy B is −1.072, 1+*A*^2^ = 1 + (0.255)^2^ = 1.065 and the Internet public opinion system |*B*| − (1 + *A*^2^) is about 0.007. Thus, the following can be introduced:


$$\begin{array}{l} |B|>1+A^{2} \end{array}$$

According to the dissipative structure criterion of the Internet public opinion diffusion process, the results reveal that the absolute value of total negative entropy of the system is greater than the absolute value of positive total entropy. Furthermore, it satisfies the dissipative structure criterion. This indicates that the system has reached a dissipative structure.

Specifically, the leading causes of the high negative total entropy of the Internet public opinion diffusion system are as follows. After public health emergencies are exposed, public anxiety, panic, and other negative emotions are constantly magnified on various social media platforms. There is an urgent need to know the truth about the event, which is a more intense demand. However, due to the limited information resources held by the relevant government departments and network media in the early stage, the information needs of the public needs cannot be met, which leads to confusion and disorder in the system. When government departments respond and deal with it effectively, much negative entropy flows quickly into the system, and the negative entropy value of the system increases rapidly.

The leading causes of the low positive total entropy of the Internet public opinion diffusion system are as follows. After the event, the relevant government departments and the media respond to the focus topic and provide effective feedback, making the truth about the event apparent and satisfy public demand. Therefore, the negative total entropy injection increases and the positive total entropy no longer accumulates.

The A1 event heat of public health emergencies and A3 net behaviors are essential sources of the positive entropy of the system and the endogenous driving force that triggers the spread of Internet public opinion. The results indicate that authoritative central media reports and government releases significantly affect the Internet public opinion negative entropy environment.

The critical point of entropy control is the criterion of whether the network public opinion system of public health emergencies has reached a dissipative structure. When the total positive entropy flow in the system is much higher than the total negative entropy flow and does not reach the point of entropy control, it indicates that the network public opinion is still in the outbreak or fluctuation period. This suggests that the relevant government departments have not yet effectively dealt with this public health emergency. The injection amount of negative entropy flow should be actively increased to promote the Internet public opinion system of public health emergencies to reach the dissipative structure. When the total positive entropy flow in the system is lower than the total negative entropy flow and has reached the point of entropy control, it indicates that the system will reach a new dissipative structure, and the network public opinion of this event will tend to the elimination stage.

## Conclusion and Implications

### Germination Mechanism: Entropy-Controlled Trigger Point

When public health emergencies are exposed, Internet-based public opinion begins to accumulate and be expressed. This period belongs to the germination stage (July 15 to 22, 2018), showing a single content and small spread. During this period, the public began to pay attention to the vaccine fraud incident but did not participate in large numbers. The whole network topic was scattered, the social platform public opinion was relatively uniform, and the vaccine-related topic forwarding and comment volume were lower. According to the comprehensive score of the Internet public opinion diffusion cycle in Table 7, the amount of negative entropy input in the germination stage is lower, promoting a higher public opinion trend. Therefore, this period is the trigger point to control the inflow of positive entropy flow of Internet public opinion and inject negative entropy flow as soon as possible.

The suggestions are as follows: the government should intervene promptly to investigate, adopt a commanding position to steer public opinion, grasp the truth about the incident as soon as possible, prepare an emergency decision-making plan, and make full use of prime time to release authoritative, comprehensive, and accurate facts to alleviate public panic and anxiety. Second, it should give full play to the ability of media agenda-setting and timely and objective reporting. Third, the two should cooperate to enhance public opinion guidance ability.

### Outbreak and Fluctuating Mechanism: Entropy-Controlled Disposal Point

During the Changchun Longevity Biotechnology Co., Ltd., vaccine fraud incident, Internet public opinion content gradually increased, becoming the whole network focus and hot spot (July 23 to August 8, 2018). The outbreak and fluctuation stage are an essential and challenging point in Internet public opinion diffusion governance. Government departments and authoritative media should give full play to controlling the positive entropy flow inflow of the system and injecting the negative entropy flow in time. On July 23, after General Secretary Jinping Xi, Prime Minister Keqiang Li, and relevant departments released information and began to deal with the incident, the focus of public opinion on the Internet continued. Social media platforms spawned other hotspots. The focus of public attention changed from denouncing and pursuing fake vaccines to questioning all domestic vaccines. The field of Internet public opinion is in a state of disorder. If the government cannot guide public opinion correctly and rationally, it will amplify the anxiety of the public and affect social stability. From the comprehensive score of the public opinion diffusion cycle in Table 7, it can be seen that the negative entropy in the fluctuation stage is the largest, and the negative entropy injection in the outbreak stage is still lower than the critical condition. Therefore, this period is the disposal point for controlling the positive entropy flow inflow of the system and injecting the negative entropy flow as soon as possible. This can preposition the entropy control point to lead a healthy and rational Internet public opinion negative entropy environment.

The suggestions are as follows. First, the relevant government departments should adopt an attitude of severe punishment toward illegal behavior and publicize the results of such punishment promptly. Second, they should establish an information feedback platform through which the media releases and provides feedback information and the targeted response to public opinion demands. Finally, they should give full play to the advantages of central media and opinion leaders to weaken the influence of untrue speech and online rumors.

### Elimination Mechanism: Latency

When vaccine fraud-related personnel at Changchun Longevity Biotechnology Co., Ltd., had been investigated, attention toward the incident decreased, and vaccine fraud-related topics began to “cool down.” Through the large-scale discussion on social network platforms such as Weibo, WeChat, and so on, the public is familiar with the development process of this public health emergency, and the panic and negative emotions of netizens have gradually been relieved. According to the comprehensive score of the Internet public opinion diffusion cycle in Table 7, the negative entropy flow is the least in the elimination stage, which indicates that the timely and effective authoritative information issued by the government promotes the rational solution of the Internet public opinion crisis. The trend of public opinion diffusion tends to be gentle. From August 9 to August 10, the “hot spot” of the incident gradually weakened, and the spread of public opinion on the entire network was controlled. Therefore, this period is the latency to control the continuous injection of negative entropy flow in the system.

The suggestions are as follows. First, to reduce the recurrence of similar events, deal with this public health emergency, and determine the crux of the problem reflected by the Internet public opinion of the incident. Second, the administrative departments of public health institutions at all levels should standardize the mechanism of issuing, monitoring, and early warning of all kinds of public health emergencies to ensure that adequate measures can be taken. This is to improve the rigor, timeliness, and authority of the relevant government departments in dealing with similar public health emergencies. Finally, we should comprehensively monitor the progress of the hot spots related to public health emergencies, control the critical nodes in the process of Internet public opinion diffusion, publish the events in time and deal with the results, and improve the execution, guidance and credibility of the government.

## Data Availability Statement

The raw data supporting the conclusions of this article will be made available by the authors, without undue reservation.

## Author Contributions

WL and WZ contributed to conception and design of the study. ZC organized the database. FZ and WL performed the statistical analysis and wrote sections of the manuscript. WZ wrote the first draft and revision of the manuscript. All authors contributed to manuscript revision, read, and approved the submitted version.

## Funding

This work was supported by National Social Science Fund of China (17BGL180), Natural Science Foundation of Hunan Province (2019JJ50277), and Scientific Research Foundation of Hunan Provincial Education Department (20B3000).

## Conflict of Interest

The authors declare that the research was conducted in the absence of any commercial or financial relationships that could be construed as a potential conflict of interest.

## Publisher's Note

All claims expressed in this article are solely those of the authors and do not necessarily represent those of their affiliated organizations, or those of the publisher, the editors and the reviewers. Any product that may be evaluated in this article, or claim that may be made by its manufacturer, is not guaranteed or endorsed by the publisher.

## References

[1] Zhang Y Chen N Du W Yao S Zheng X. A New geo-propagation model of event evolution chain based on public opinion and epidemic coupling. Int J Environ Res Public Health. (2020) 17:9235. 10.3390/ijerph17249235 33321897 PMC7764303

[2] Giles EL Adams JM. Capturing public opinion on public health topics: a comparison of experiences from a systematic review, focus group study, and analysis of online, user-generated content. Front Public Health. (2015) 3:200. 10.3389/fpubh.2015.00200 26380248 PMC4547000

[3] Dai W Hu H Wu T Dai Y. Information spread of emergency events: path searching on social networks. ScientificWorldJournal. (2014) 2014:179620. 10.1155/2014/179620 24600323 PMC3926277

[4] Han X Wang J Zhang M Wang X. Using social media to mine and analyze public opinion related to COVID-19 in China. Int J Environ Res Public Health. (2020) 17:2788. 10.3390/ijerph17082788 32316647 PMC7215577

[5] Jo W Lee J Park J Kim Y. Online information exchange and anxiety spread in the early stage of the novel coronavirus (COVID-19) outbreak in South Korea: structural topic model and network analysis. J Med Internet Res. (2020) 22:e19455. 10.2196/19455 32463367 PMC7268668

[6] Xiong J Hswen Y Naslund JA. Digital surveillance for monitoring environmental health threats: a case study capturing public opinion from twitter about the 2019 Chennai water crisis. Int J Environ Res Public Health. (2020) 17:5077. 10.3390/ijerph17145077 32674441 PMC7400361

[7] He W Wang FK Akula V. Managing extracted knowledge from big social media data for business decision making. J Knowl Manage. (2017) 21:275–94. 10.1108/JKM-07-2015-0296

[8] China Internet Network Information Centre (CNNIC). The 44^th^ Statistical Report on Internet Development in China. Available online at: https://cnnic.com.cn/IDR/ReportDownloads/201911/P020191112539794960687.pdf

[9] Brent BS. Prigogine's model for self-organization in non-equilibrium systems. Human Dev. (1978) 21:374–87. 10.1159/000272417

[10] Allen PM. Self-organization and dissipative structures. Techn Phys Lett. (1990) 16:248–51.

[11] Kåberger T Månsson B. Entropy and economic processes - physics perspectives. Ecol Econ. (2001) 36:165–79. 10.1016/S0921-8009(00)00225-1

[12] Prigogine I. Symmetry breaking instabilities in dissipative systems II. J Chem Phys. (1968) 48:1695–700.

[13] Yu YH Lv F Tang CH. The management entropy research of enterprise network based on brusselator model. J. Business Econ. (2014) 1:34–41.

[14] Deng X Zheng S Xu P Zhang X. Study on dissipative structure of China's building energy service industry system based on brusselator model. J Clean Product. (2017) 150:112–22. 10.1016/j.jclepro.2017.02.198

[15] Fink S. Crisis Management: Planning for the Inevitable. New York, NY: American Management Association (1986).

[16] Pauchant TC Mitroff II. Transforming the Crisis-Prone Organization. San Francisco: Jossey-Bass (1992).

[17] Coombs WT. Ongoing Crisis Communication. Thousand Oaks, CA: Sage (1999).

[18] He W Fang Y Malekian R Li Z. Time series analysis of online public opinions in colleges and universities and its sustainability. Sustainability. (2019) 11:3546. 10.3390/su11133546

[19] Zhao Y Cheng S Yu X Xu H. Chinese public's attention to the COVID-19 epidemic on social media: observational descriptive study. J Med Internet Res. (2020) 22:e18825. 10.2196/18825 32314976 PMC7199804

